# Using emotion to guide decisions: the accuracy and perceived value of emotional intensity forecasts

**DOI:** 10.1007/s11031-023-10007-4

**Published:** 2023-03-30

**Authors:** Steven J. Carlson, Linda J. Levine, Heather C. Lench, Elinor Flynn, Kaitlin M. H. Winks, Britanny E. Winckler

**Affiliations:** 1 Department of Psychological Science, University of California, Irvine, USA; 2 Department of Psychological and Brain Sciences, Texas A&M University, College Station, USA; 3 Management Department, The Wharton School, University of Pennsylvania, Philadelphia, USA; 4 Division of Hospital Medicine, Children’s Hospital of Orange County, Orange, CA USA

**Keywords:** Affective forecasting, Emotion, Intensity, Frequency, Duration, Decision making

## Abstract

Forecasts about future emotion are often inaccurate, so why do people rely on them to make decisions? People may forecast some features of their emotional experience better than others, and they may report relying on forecasts that are more accurate to make decisions. To test this, four studies assessed the features of emotion people reported forecasting to make decisions about their careers, education, politics, and health. In Study 1, graduating medical students reported relying more on forecast emotional intensity than frequency or duration to decide how to rank residency programs as part of the process of being matched with a program. Similarly, participants reported relying more on forecast emotional intensity than frequency or duration to decide which universities to apply to (Study 2), which presidential candidate to vote for (Study 3), and whether to travel as Covid-19 rates declined (Study 4). Studies 1 and 3 also assessed forecasting accuracy. Participants forecast emotional intensity more accurately than frequency or duration. People make better decisions when they can anticipate the future. Thus, people’s reports of relying on forecast emotional intensity to guide life-changing decisions, and the greater accuracy of these forecasts, provide important new evidence of the adaptive value of affective forecasts.

## Introduction

People rely on affective forecasts – judgments about how future outcomes will make them feel – to make decisions ranging from whether to take a vacation (Wirtz et al., 2003) to which medical procedures to undergo to treat cancer (Perry et al., 2020). They pour effort and resources into achieving outcomes that they predict will make them happy and into avoiding outcomes that they predict will make them miserable (Mellers & McGraw, 2001). But decades of research show that people are poor at predicting how they will feel (e.g., Gilbert et al., 1998, 2002; Kahneman et al., 2006), and that inaccurate forecasts can impair decision making (e.g., Dorison et al., 2019; Halpern & Arnold, 2008). Why would people base decisions on judgments that are so often faulty? To better understand the adaptive value of affective forecasts, we examined whether people forecast some features of their future emotional experience more accurately than others, and whether they report basing decisions primarily on the forecasts that are more accurate.

Central to this investigation is the recognition that emotional experience is dynamic. Just as a musical phrase in a song can be loud or soft, occur more or less frequently, and last for a longer or shorter period of time, emotions vary in their intensity, frequency, and duration. The intensity, or strength, of a person’s emotional response to an event depends chiefly on the importance of the event for the person’s goals (e.g., Sonnemans & Frijda, 1995). Emotion frequency and duration also depend on event importance. However, frequency – how often an emotion occurs – also depends on thoughts or external cues that bring the event to mind. Duration – how long an emotion lasts before returning to a baseline or neutral affective state – also depends on emotion regulation strategies such as rumination or reappraisal (Verduyn & Lavrijsen, 2015).

The dynamic nature of emotional experience raises important questions about affective forecasting. First, when people try to anticipate how the outcomes of their decisions will make them feel, which features of their future emotional experience do they bring to mind? For example, to decide which career to pursue, do people report anticipating how *intensely* happy they will feel in the profession, how *frequently* they will feel happy, or how *long* their happy feelings will last? Second, are people better at forecasting some features of emotion than others? Thus, to better understand how affective forecasts contribute to decision making, we assessed how much people reported relying on forecasts of emotional intensity, frequency, and duration to make major decisions. We also assessed how accurately they forecast each emotion feature, and whether forecasting accuracy predicted satisfaction with decisions.

### The accuracy of forecasts of specific features of emotion

It is widely accepted that people are poor at predicting how they will feel in the future and often overestimate the emotional impact of events, a tendency known as the impact bias (Gilbert et al., 2002). People overestimate how long they will suffer after the break-up of a romantic relationship (Gilbert et al., 1998), how happy a higher income will make them (Kahneman et al., 2006), and how much they will enjoy their vacations (Wirtz et al., 2003). They overestimate because, when anticipating how a future event will make them feel, people focus on the event’s most salient, central aspects (e.g., watching the sun set over the beach on vacation). They neglect to consider more peripheral aspects (sunburn, wet sandy clothes) that will also occupy their attention and mitigate their emotional response (Wilson et al., 2000). People also fail to anticipate how quickly they will adapt to events (Gilbert et al., 1998). Thus, focusing on the central aspects of events and failing to consider adaptation both contribute to overestimating emotion.

Importantly, however, forecasting accuracy varies across emotion features. A meta-analysis and experimental studies showed that people are more accurate when they forecast the intensity of their feelings about an event than when they forecast how they will feel “in general”, a judgment commonly assessed in the forecasting literature (Levine et al., 2012; also see Doré et al., 2016). Lench and colleagues (2019) examined affective forecasts about an election outcome, receiving an exam grade, and losing money in a laboratory task. Across studies, participants forecast the intensity of their emotional response with high accuracy but overestimated the frequency of emotion and the impact of events on their overall mood. Thus, although emotional intensity can be overestimated (Charpentier et al., 2016) or underestimated (Lench et al., 2011), the few studies that have directly compared forecasting accuracy across features show that people forecast emotional intensity relatively accurately.

Several factors may promote greater accuracy in forecasts of emotional intensity than frequency or duration. When forecasting how they will feel, people focus on the most central, goal-relevant, aspects of future events (Wilson et al., 2000). Similarly, when experiencing intense emotion, people’s attention narrows to central, goal-relevant aspects of events at the expense of peripheral details (Levine & Edelstein, 2009). This common focus of attention when people forecast and experience the peak intensity of emotion helps to explain why they forecast intensity relatively accurately. In contrast, the frequency and duration of emotion depend, not only on the importance of events for a person’s goals, but also on thoughts and regulatory processes that shift rapidly over time, depend on concurrent events, and are harder to anticipate (Lench et al., 2019; Levine et al., 2012).

### Reported reliance on forecasts of specific features of emotion to make decisions

The relative accuracy with which people forecast emotional intensity may bode well for their decisions. But that depends, of course, on whether they base their decisions on forecast intensity. There are compelling reasons to privilege forecasts of emotional intensity when making decisions. First, as noted above, intensity forecasts tend to be relatively accurate (Doré et al., 2016; Lench et al., 2019; Levine et al., 2012). Second, these forecasts may be particularly salient and come easily to mind. Past research shows that peak intensity plays a critical role in people’s global evaluation of an experience (e.g., a film, a medical procedure), with duration playing a negligible role (Fredrickson, 2000; Kahneman et al., 1993). Third, intensity forecasts are informative. Emotional intensity signals how closely an outcome aligns with people’s goals and the importance of those goals (e.g., Frijda et al., 1992). Thus, to make decisions, people may rely more on forecasts of emotional intensity than frequency or duration because this feature best conveys how important an outcome will be and the amount of effort it is worth expending to achieve or avoid it (Fredrickson, 2000).

Yet there are also compelling reasons for people to base decisions on forecasts of the frequency or duration of emotion. Experience sampling studies show that overall life satisfaction is more highly correlated with the frequency with which people report feeling happy than with the intensity of their happiness (Diener et al., 2009; Jachimowicz et al., 2021). Whereas the intensity of emotion reflects the importance of an event while it is the current focus of attention, the frequency and duration of emotion reflect the importance of an event in the context of other ongoing events, thoughts, and priorities. In other words, emotion frequency and duration give an indication of how much an event impacts a person’s daily life (Diener et al., 2009; Verduyn et al., 2015). Thus, when making decisions with lasting consequences, such as selecting careers, educational programs, or health behaviors, people may consider the peak intensity of future emotion to be a fleeting experience that matters less for their future satisfaction than the frequency or duration of emotion. To our knowledge, however, no studies have examined the features of emotion people report forecasting to make important decisions. Nor have they compared the extent to which accuracy in forecasting emotional intensity, frequency, and duration predicts later satisfaction with decisions.

Finally, it is not known how much people report relying on forecast happiness versus unhappiness to make decisions. People tend be unrealistically optimistic when anticipating the future (Barsics et al., 2016; Lench et al., 2021). They anticipate that the Netflix movie will be enjoyable, their career successful, and their children agreeable. This optimistic bias persists even when the outcome is uncontrollable and equally likely to be positive or negative (Sharot, 2011). These findings suggest that, to make decisions, people may focus more on how happy positive outcomes will make them than on how unhappy negative outcomes will make them.

### The current investigation

In four studies, we investigated how much participants reported relying on forecasts of the intensity, frequency, and duration of happiness and unhappiness to make decisions about their careers, education, politics, and health. Two of these studies also assessed the accuracy with which participants forecast the intensity, frequency, and duration of emotion. In Study 1, a three-part longitudinal study, we assessed how much graduating medical students reported having relied on forecasts of the intensity, frequency, and duration of happiness and unhappiness to decide how to rank residency programs prior to being matched with a program. We also assessed the accuracy with which students forecast the intensity, frequency, and duration of happiness they would feel when they learned which program they would attend. One way of assessing the value of forecasts is to find out whether people are satisfied with the outcomes of their decisions. Therefore, three months into their residency programs, we assessed satisfaction with the program. Few studies of affective forecasting track the outcomes of real-world, life-changing decisions – a valuable contribution of this study.

We conducted three follow-up studies to determine how much people report relying on forecast emotional intensity, frequency, and duration to make decisions in other important domains: education, politics, and health. Each study built on the previous studies in important ways. To decide among educational programs, people may forecast how they will feel when they learn which program they will attend, as assessed in Study 1. People may also forecast how they will feel during the program itself. Thus, in Study 2, we asked participants how much they had relied on forecasts of the intensity, frequency, and duration of their feelings *as a student* in a college to decide which colleges to apply to. Building on Studies 1 and 2, which concerned deciding among positive outcomes (residency programs, colleges), Study 3 assessed how people decided which candidate to vote for in a presidential election – a choice often motivated by the desire to avoid negative outcomes (Finkel et al., 2020). Study 3 also extended prior studies by assessing how accurately participants forecast each feature of both negative and positive emotion about the election outcome. Finally, the first three studies assessed participants’ reports of how they made decisions in the recent past (e.g., ranking residency programs). This timing allowed participants to reflect on how they made their choices and avoided ethical concerns that study questions might influence their choices. However, self-reports about past decisions may be subject to retrospective biases (Nisbett & Wilson, 1977). Therefore, in Study 4, we asked participants how they would make a *future* decision about whether to travel during the Covid-19 pandemic.

In summary, affective forecasts are often inaccurate so it is puzzling that people rely on them to make life-changing decisions (Perry et al., 2020). We hypothesized that people would report basing their decisions primarily on forecasts that tend to be more accurate. Because emotional intensity is a highly salient signal of the importance and goal relevance of events (e.g., Frijda et al., 1992; Fredrickson, 2000), we expected participants to report relying more on forecast intensity than frequency or duration to make decisions. We further expected participants to forecast emotional intensity more accurately than emotion frequency or duration (Lench et al., 2019).

**Transparency and openness.** Data and software code for all four studies are publicly available at the Open Science Framework (OSF) and can be accessed at https://osf.io/3548z/. Study 4 hypotheses were preregistered at: https://aspredicted.org/CMQ_CMV. For all studies, we report any data exclusions and provide the precise wording of instructions and questions.

## Study 1

During their final year of medical school, students undergo a competitive process known as “the Match”. The result determines where students will receive three to seven years of residency training, setting the stage for the type of career they will have as physicians and significantly impacting their personal lives. After applying to programs and completing interviews, medical students rank order their most favored residency programs. Their ranking decisions have an impact on whether they match and the specific program to which they are matched. Programs also rank order applicants. This information is sent to a centralized service that matches students to residency programs. Students who are successful in matching find out which residency program they were matched with on “Match Day” – the third Friday of March – and are obligated to attend that program (Curtin & Signer, 2017).

In Study 1, graduating medical students completed three online questionnaires. At Time 1, days after the deadline for submitting their ranked list of residency programs, but before they learned the program they were matched with, medical students reported how much they had relied on forecasts of the intensity, frequency, and duration of happiness and unhappiness to decide how to rank residency programs. Two considerations, one practical and one ethical, determined the timing of this assessment. Practically, having recently submitted their rank order lists allowed medical students to reflect on how they had made their choices. Ethically, the timing ensured that study questions did not influence how medical students went about making potentially life-changing ranking decisions.

Participants then forecast the intensity, frequency, and duration of happiness they would feel during an evening the week after Match Day if they matched with different programs on their list. At Time 2, an evening the week after Match Day, medical students reported the intensity, frequency, and duration of happiness they were experiencing. Thus, participants forecast their happiness (Time 1) and reported their experienced happiness (Time 2) for the same time period: an evening, the week after Match Day. This allowed us to assess the accuracy of participants’ forecasts of the intensity, frequency, and duration of happiness they would experience during a specific, discrete period of time. People’s ability to introspect about feelings and decision making processes is limited (Nisbett & Wilson, 1977). To find out whether participants made distinctions among features of emotion, we also examined the proportion of variance in their reports of each feature that could not be attributed to the other features. Finally, months after beginning their residency programs (Time 3), participants rated their satisfaction with their programs.

### Method

Study 1 was part of a larger investigation of decision-making that assessed other aspects of medical student’s forecasts, emotions, decisions, prior training, and appraisals, which were not the focus of this paper (Kaiser et al., 2022; Lench et al., 2019, 2021).

**Participants.** Students (*N* = 178) completing their fourth year of medical school at a large public university in California participated in the study. All fourth-year students in two sequential cohorts, who were participating in Match Day in March, were invited to take part. Participants received $25 for completing the first survey, and $50 each for the second and third surveys. From an initial sample of 204 students, 182 students agreed to participate. We excluded data from four students who did not complete all three questionnaires, one of whom indicated not having matched.

Prior to conducting the study, we planned to collect data from 200 participants, based on estimated enrollment rates across two years in the medical program, acknowledging that the availability of this sample would limit power. A post hoc power analysis using G*Power (Faul et al., 2007) indicated that, for an ANOVA on reliance ratings, testing a 3 (emotion feature) x 2 (valence) interaction between within-subject factors, the final sample provided 84% power at 0.05 to detect a small effect (Cohen’s *f*^*2*^ = 0.10). Our interest in detecting a small effect was based on prior research showing that ratings of emotional intensity, frequency, and duration are typically correlated because all three features depend in part on the importance of an outcome for a person’s goals (Sonnemans & Frijda, 1995; Verduyn & Lavrijsen, 2015). This overlap would reduce the likelihood of observing large differences among features. Detecting a difference with a small effect size in reported reliance on emotion features would demonstrate that, although related, people distinguish among features and perceive differences in how much they rely on specific features to make decisions.

Participants were men (47%) and women (53%) whose ages ranged from 25 to 36 years, *M* = 28.02 years. Participants reported their ethnicity as White (39%), East Asian (17%), Latino/a (13%), South Asian (11%), Middle Eastern (8%), Pacific Islander (2%), Black (2%), or other (8%). The research was carried out in accordance with the University’s Institutional Review Board (IRB).

**Procedure and materials: Time 1 questionnaire – before Match Day.** Fourth year medical students completed three online questionnaires. They were emailed a link to the first questionnaire the last week of February, a few days after the deadline for submitting their rankings of residency programs to the National Resident Matching Program. They completed the questionnaire by the first week of March, a week before Match Day.

*Rank Order List (ROL).* Participants indicated the residency programs they ranked first through fourth on their ROL and the specialty area of each program (e.g., neurology).

*Reported reliance on forecasts of different emotion features.* Participants rated how much they had relied on forecasts of different features of emotion to rank residency programs. They were instructed, “To help them make difficult decisions, people may try to predict their future emotional experience – how the outcome of their decisions will make them feel. Emotional experience, like music, has several features. For instance, a particular musical note in a song can be gentle or strong (intensity), short or long (duration), and occur rarely or often (frequency). When you were deciding whether to rank a program highly on your ROL, how important were your predictions about these features of your future emotional experience?” Participants rated how much they had relied on forecasts concerning the intensity, duration, and frequency of happiness and unhappiness, using a scale from 1 (*not at all important when I made my decision*) to 9 (*extremely important when I made my decision*). Specifically, they rated, “How happy I’d feel if I match with that program (intensity),” “How long I’d feel happy if I match with that program (duration),” “How often I’d feel happy if I match with that program (frequency),” “How unhappy I’d feel if I do NOT match with that program (intensity),” “How long I’d feel unhappy if I do NOT match with that program (duration),” “How often I’d feel unhappy if I do NOT match with that program (frequency).”

*Forecast emotion.* Participants then forecast the intensity, frequency, and duration of happiness they would feel if they were matched with the program they had ranked first, second, third, and fourth or lower. We assessed forecast intensity, using a scale from 1 (*not at all*) to 9 (*most extreme possible*), by asking, “Suppose it’s an evening during the week after Match Day, and you matched with the program you ranked [first / second / third / fourth or lower]. How will you be feeling about matching with that residency program? How intensely will you feel happy?” We assessed forecast frequency, using a scale from 1 (*never*) to 9 (*constantly*), by asking, “How frequently that day will you feel happy about matching with the residency program you ranked [first / second / third / fourth or lower]?” We assessed forecast duration of mood by asking, “Overall, how much of the time that day will you spend in a mood that is happy?” Participants responded using an 11-point scale that increased in increments of 10%, from 0 (*not at all*) to 10 (*100% - the entire day*). Given the length of the questionnaire, and the need to assess forecast intensity, frequency, and duration for each of four ranked programs, this study did not assess forecast unhappiness.

**Time 2 questionnaire: After Match Day**. Participants received a link to a second online questionnaire the day after Match Day and completed it during an evening within a week. They reported the program to which they had been matched, where they had ranked that program on their list, and whether they considered the match to be a positive or negative outcome. Participants then reported their emotional experience using the same scales they had used for emotion forecasts. Specifically, we assessed experienced intensity by asking, “You matched with the residency program you ranked [first / second / third / fourth or lower]. How are you feeling about matching with that residency program? How intensely are you feeling happy?” We assessed experienced frequency by asking, “How frequently today did you feel happy about matching with your residency program?” We assessed experienced duration of mood by asking, “Overall, how much of the time today did you spend in a mood that was happy?”

**Time 3 questionnaire: During residency.** In October, about six months after Match Day and three months after beginning their residency program, participants rated how satisfied they were with their program, from 1 (*not at all satisfied*) to 9 (*most satisfied possible*).

**Analytic plan.** We conducted separate ANOVAs to assess how much participants reported relying on different features of emotion to make decisions, and to assess how accurately participants forecast different features of emotion. Ratings that are made by the same individual may be correlated. Therefore, ANOVAs were conducted using the SAS GLM procedure, with the Repeated statement, which takes into account the within-subject correlations that result when multiple ratings are made by each participant (SAS Institute, 2017). In addition, we report *Cohen’s d*_*Repeated Measures, pooled *_*(d*_*RM, pool*_*)* for effect sizes for post hoc within-subject comparisons, which controls for the correlations among measurements (Lakens, 2013). Although participants provided multiple ratings in all four studies, all within-subject variables were crossed with each other, and with any between-subjects variables (rather than nested). Finally, because the number of participants varied by group in some studies (e.g., in Study 1, more participants matched with their first ranked program than with lower ranked programs), we used Type III Sums of Squares to test for significant effects in ANOVAs in all studies. This approach is conservative and is commonly recommended for testing effects in unbalanced designs that may include significant interactions (Smith & Cribbie, 2014).

### Results and discussion

All but one of the participants was matched with a residency program: 52% matched with their first ranked program, 19% with their second ranked program, 10% with their third ranked program, and 19% with a program ranked fourth or lower. The week after their match, 95% of the participants considered their match to be a positive rather than negative outcome.

**Fig. 1:**
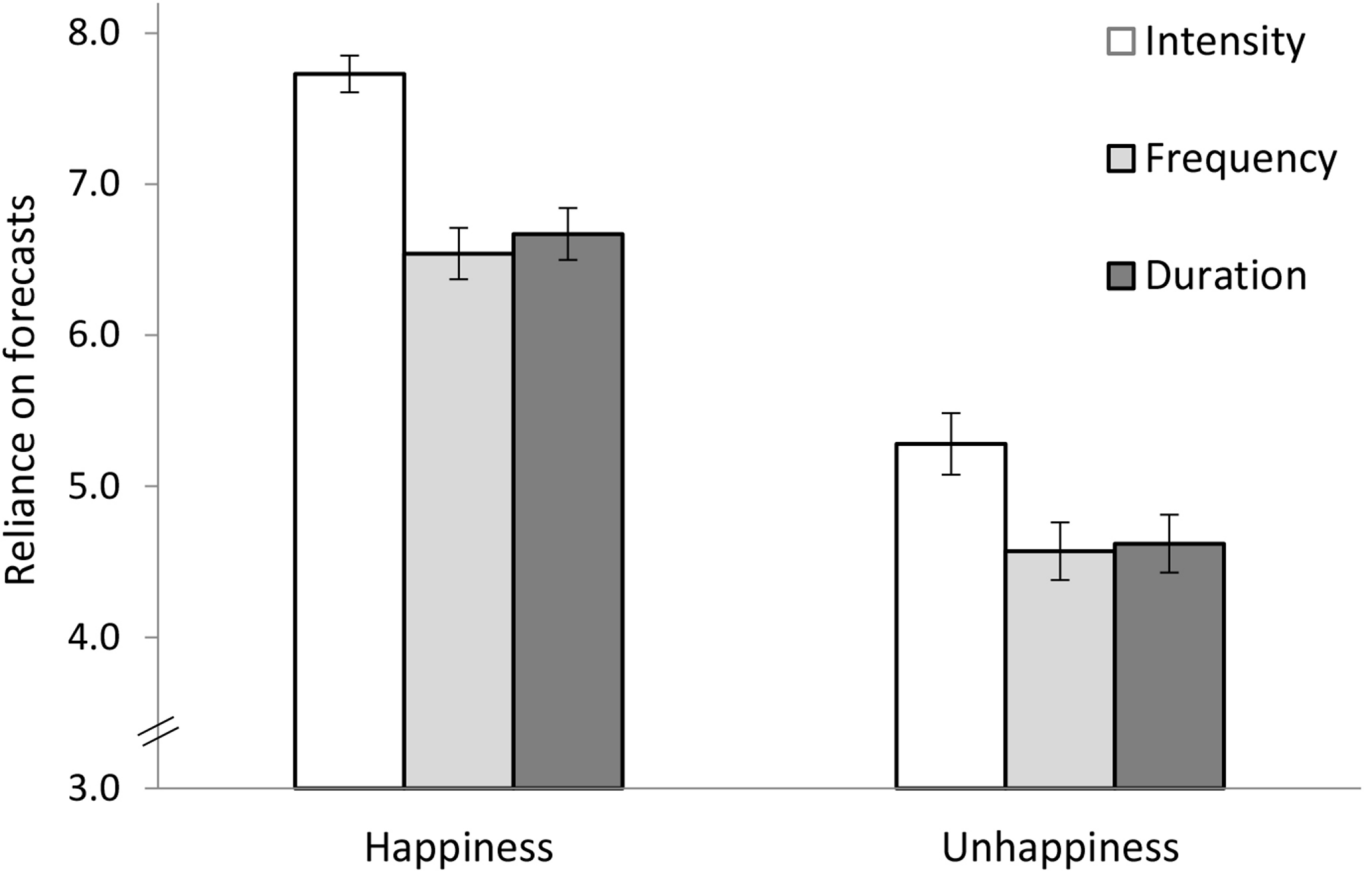
Medical Students’ Reports of How Much They Relied on Forecasts of Specific Features of Emotion to Rank Residency Programs (Study 1) *Note.* Error bars represent ± 1 standard error

**Reported reliance on forecasts of specific features of emotion to rank programs.** Figure 1 shows how much participants reported having relied on forecasts of different features of happiness and unhappiness to rank residency programs. We conducted a repeated measures ANOVA on participants’ reliance ratings with emotion feature (intensity, frequency, duration) and valence (happiness, unhappiness) as within-subject variables. The results showed a significant effect of emotion feature, *F*(2, 348) = 45.92, *MSE* = 98.17, *p* < .001, η^2^_*p*_ = 0.21. As Fig. 1 shows, participants reported having relied more on forecasts of the intensity of emotion to rank programs than on forecasts of the frequency or duration of emotion. Specifically, they reported having relied more on forecast intensity than frequency, both for happiness, *t*(176) = 7.36, *p* < .001, *d*_*RM, pool*_ = 0.52, 95% CI [0.87, 1.50], and unhappiness, *t*(176) = 5.61, *p* < .001, *d*_*RM, pool*_ = 0.65, 95% CI [0.46, 0.97]. They reported having relied more on forecast intensity than duration, both for happiness, *t*(177) = 6.40, *p* < .001, *d*_*RM, pool*_ = 0.44, 95% CI [0.73, 1.38], and unhappiness, *t*(175) = 5.20, *p* < .001, *d*_*RM, pool*_ = 0.60, 95% CI [0.42, 0.93]. Reported reliance on forecast frequency and duration did not differ for happiness, *t*(176) = -1.01, *p* = .31, *d*_*RM, pool*_ = 0.11, 95% CI [-0.37, 0.12], or unhappiness, *t*(176) = -0.86, *p* = .39, *d*_*RM, pool*_ = 0.13, 95% CI [-0.20, 0.08]. (CIs for all *t*-tests are given for the difference between means.)

The results also showed a significant main effect of valence, *F*(1, 174) = 155.78, *MSE* = 1224.72, *p* < .001, η^2^_*p*_ = 0.47. To rank programs, participants reported having relied more on forecasts of how happy they would feel if they matched with a program than on how unhappy they would feel if they did not match with a program. Finally, an interaction between feature and valence was found, *F*(2, 348) = 4.89, *MSE* = 5.01, *p* = .008, η^2^_*p*_ = 0.03. Although participants reported having relied significantly more on forecast intensity than frequency or duration for both happiness and unhappiness, this difference was more pronounced for happiness than unhappiness.

### Forecasting accuracy

Medical students reported the intensity, frequency, and duration of happiness they forecast, and later experienced, an evening during the week after Match Day, if they matched with different programs on their list. We compared the accuracy of participants’ forecasts by examining overall inaccuracy. We selected this approach because the average direction of forecasting bias (i.e., forecast minus experienced emotion) masks overall inaccuracy if some participants overestimate and others underestimate. Therefore, we assessed the overall inaccuracy of forecasts, independent of the direction of bias, by computing the absolute value of the difference between forecast and experienced emotion. Lower values indicate greater accuracy (i.e., less difference between forecasts and experience). We conducted a mixed model ANOVA on overall inaccuracy. The within-subject factor was emotion feature (intensity, frequency, duration). To find out whether participants were more inaccurate when forecasting their emotional response to more disliked outcomes, the between-subjects factor was participants’ ranking of the residency programs to which they were matched (1st choice, 2nd choice, 3rd choice, 4th or lower choice).

The results showed a main effect of feature, *F*(2, 352) = 9.31, *MSE* = 11.22, *p* < .001, η^2^_*p*_ = 0.05. As lower values indicate greater accuracy, participants forecast the intensity of happiness (*M* = 0.89, *SD* = 1.08) more accurately than the frequency of happiness (*M* = 1.12, *SD* = 1.19), *t*(177) = 2.57, *p* = .01, *d*_*RM, pool*_ = 0.18, 95% CI [0.05, 0.42], and the duration of happiness (*M* = 1.62, *SD* = 1.62), *t*(177) = 5.98, *p* < .001, *d*_*RM, pool*_ = 0.38, 95% CI [0.49, 0.98]. They also forecast the frequency of happiness more accurately than the duration of happiness, *t*(177) = 3.81, *p* < .001, *d*_*RM, pool*_ = 0.23, 95% CI [0.24, 0.76]. A main effect of program rank was also found, *F*(1, 176) = 25.60, *MSE* = 62.77, *p* < .001, η^2^_*p*_ = 0.13. We computed correlations between program rank and forecasting accuracy for each emotion feature. Participants forecast their reactions to matching with programs that they had ranked lower more inaccurately for all three emotion features: intensity, *r*(178) = 0.43, *p* < .001; frequency, *r*(178) = 0.21, *p* = .005; duration, *r*(178) = 0.20, *p* = .007.

We also assessed the direction of bias – the extent to which participants over- or underestimated in forecasting how they would feel after Match Day. For the intensity, frequency, and duration of happiness, we conducted paired *t*-tests comparing forecasts to experience. No significant bias was found for forecasts of the intensity of happiness (forecast: *M* = 7.64, *SD* = 1.56; experience: *M* = 7.53, *SD* = 1.72; *t*(177) = 1.07, *p* = .28, *d*_*RM, pool*_ = 0.10), but participants overestimated how frequently they would feel happy (forecast: *M* = 7.27, *SD* = 1.57; experience: *M* = 7.00, *SD* = 1.84; *t*(177) = 2.22, *p* = .03, *d*_*RM, pool*_ = 0.18), and how long their happiness would last (forecast: *M* = 6.93, *SD* = 2.15; experience: *M* = 6.21, *SD* = 2.60; *t*(177) = 4.36, *p* < .001, *d*_*RM, pool*_ = 0.36).

**Satisfaction with residency programs.** People engage in affective forecasting to make decisions with satisfying outcomes. We examined whether the accuracy with which medical students forecast different emotion features predicted how satisfied they were with their program months later during residency. To find out whether accuracy in forecasting intensity, frequency, or duration made a unique contribution to satisfaction, we conducted a regression analysis that included forecasting accuracy for all three features, and program rank, as predictors.

The regression model predicting participants’ satisfaction with their programs during residency was significant, *R*^*2*^ = 0.08, *F*(4, 161) = 3.55, *MSE* = 6.59, *p* = .008. As a reminder, we computed the absolute value of the difference between forecast and experienced happiness. Thus, lower values indicate greater accuracy. Greater accuracy in forecasting emotional intensity uniquely predicted greater satisfaction with the program during residency, *β =* − 0.28, *b* = − 0.36, *SE* = 0.12, *t* = -2.99, *p* < .001. Surprisingly, *inaccuracy* in forecasting the frequency of happiness predicted greater satisfaction, *β =* 0.19, *b* = 0.23, *SE* = 0.10, *t* = 2.24, *p* = .03. Satisfaction was not predicted by the accuracy of duration forecasts, *β = − .1*0, *b* = − 0.09, *SE* = 0.07, *t* = -1.31, *p* = .19, or program rank, *β =* 0.06, *b* = 0.08, *SE* = 0.10, *t* = 0.75, *p* = .45.

**Differentiation of emotion features.** Finally, given the limitations in people’s introspective abilities, it was important to assess whether participants could actually differentiate among specific features of emotion. We examined correlations among participants reports of the intensity, frequency, and duration of happiness for three emotion judgments: reliance on forecast happiness to make decisions, forecast happiness, and experienced happiness. This allowed us to determine the proportion of variance in reports of one feature that could not be attributed to the other features.

First, we examined participants’ reports of reliance on forecasts of different features of happiness. Correlations among features were moderate, with an average correlation of 0.55: *r*_int*freq_ = 0.43, 95% CI [0.30, 0.54]; *r*_int*dur_ = 0.41, 95% CI [0.28, 0.52]; *r*_freq*dur_ = 0.74, 95% CI [0.67, 0.80]. The average coefficient of determination (i.e., *R*^2^, the proportion of variance in reports of one feature that could be attributed to the other features) was approximately 0.30. Thus, an average of 70% of the variance in reliance on each feature was not explained by the other two features. Second, we examined forecast happiness about the residency programs to which participants were matched. Correlations among features were moderate to high: *r*_int*freq_ = 0.84, 95% CI [0.79, 0.88]; *r*_int*dur_ = 0.63, 95% CI [0.53, 0.71]; *r*_freq*dur_ = 0.67, 95% CI [0.58, 0.75]. The average correlation between features was 0.73, and *R*^2^ = 0.53. Thus, on average, 47% of the variance in reports of one emotion feature was not explained by the other two features. Third, for experienced happiness, correlations among features were also moderate to high: *r*_int*freq_ = 0.71, 95% CI [0.62, 0.77]; *r*_int*dur_ = 0.70, 95% CI [0.61, 0.76]; *r*_freq*dur_ = 0.66, 95% CI [0.57, 0.73]. The average correlation among features was 0.69, and *R*^2^ = 0.47. On average, 53% of the variance in reports of one emotion feature was not explained by the other two features.

Taken together these findings showed that, as would be expected, participants’ reports of emotional intensity, frequency, and duration were correlated. However, the findings also provide evidence that participants made distinctions among emotional intensity, frequency, and duration, with approximately 47–70% of variance in reports of each feature of emotion not explained by the other two features.

In summary, graduating medical students reported having relied more on forecast emotional intensity than frequency or duration to decide how to rank residency programs. They also forecast the intensity of happiness they would feel after Match Day more accurately than they forecast the frequency or duration of happiness. Indeed, analyses of the direction of bias showed no significant bias in forecasts of the intensity of happiness. In contrast, participants overestimated the frequency and duration of happiness (also see Lench et al., 2019). Participants also showed more overall inaccuracy when forecasting their emotional response to matching with programs they had ranked lower. This is consistent with prior findings of greater forecasting inaccuracy for negative than positive outcomes (e.g., Gilbert et al., 1998).

Importantly, the more accurately medical students forecast how intensely happy they would feel about matching with their residency program, the more satisfied they were months into their residency program, even after taking into account the accuracy with which they forecast the frequency and duration of happiness and the rank of the program they matched with. Accurately forecasting intensity may predict greater satisfaction because emotional intensity encapsulates a wealth of self-relevant information including the significance of an outcome for the achievement of a person’s goals (Fredrickson, 2000; Frijda et al., 1992). Unexpectedly, the *less* accurately participants forecast the frequency of their happiness, the more satisfied they were with their residency programs. While unexpected, this finding is consistent with the view that inaccurate forecasts can be motivating (Morewedge & Buechel, 2013). Optimistic individuals tend to forecast that they will feel happy and report being satisfied with their lives (Lench et al., 2021). Thus, more optimistic participants may have overestimated more when forecasting the frequency of happiness and also experienced greater satisfaction with their residency programs.

### Follow-up studies

We conducted three follow-up studies to find out whether separate groups of participants reported relying more on forecast emotional intensity than frequency or duration to make decisions in other important domains: education, politics, and health. We also further explored the extent to which people rely on forecast happiness versus unhappiness to make decisions. Study 2 examined decisions that involved selecting among positive outcomes: deciding which universities to apply to. Study 3 examined decisions partly motivated by the desire to avoid negative outcomes: deciding which presidential candidate to vote for. Study 4 examined decisions about outcomes that were ambiguous: deciding whether to travel as rates of Covid-19 were declining. These studies also assessed both how participants made decisions in the past (Studies 2 and 3) and how they were making decisions for the future (Study 4). Finally, whereas Study 1 assessed how accurately medical students forecast features of happiness, Study 3 assessed forecasting accuracy for features of negative and positive emotion.

## Study 2

To decide among programs, people may forecast their emotional response to learning which program they will attend. We examined these forecasts in Study 1. People may also decide among programs by forecasting how they will feel over the course of a program. Thus, building on Study 1, Study 2 assessed participants’ forecasts of how they would feel as a student in college. Specifically, participants were asked how much they had relied on forecasts of the intensity, frequency, and duration of their feelings as a student at a college in order to decide which colleges to apply to.

### Method

**Participants.** As part of a larger investigation on decision making, undergraduates (*N* = 404) from a public university in California completed an online questionnaire after an unrelated experiment. All undergraduates who signed up via the campus research subject pool in the academic term were invited to participate and compensated with course credit. We excluded data from four participants who indicated they did not wish their data to be used. As part of the larger investigation, we conducted an a priori G*power analysis for the difference between two dependent means. The results gave a total sample size of 327 to have power of 0.95 to detect an effect size of 0.20. Prior research shows that ratings of emotion intensity, frequency, and duration tend to be correlated (Sonnemans & Frijda, 1995; Verduyn & Lavrijsen, 2015). Based on these findings, we also viewed having power to detect a small effect size as appropriate for comparisons of how much participants reported relying on different features of emotion to make decisions. Participants were women (81%) and men (19%) who reported their ethnicity as Latino/a (28%), East Asian (27%), White (15%), Southeast Asian (14%), or other (16%). The research was carried out in accordance with the University’s IRB.

**Procedure and materials.** Participants rated how much they relied on forecasts of six features of emotion to decide which colleges to apply to. They were instructed: “To help them make difficult decisions, people may try to predict their future emotional experience – how the outcome of their decisions will make them feel. When you were deciding which universities to apply to for your college education, how important were your predictions about these features of your future emotional experience?” Participants rated reliance from 1 *(not at all important when I made my decision)* to 9 *(extremely important when I made my decision)*. They first rated reliance on forecast happiness: “How happy I’d feel as a student at that college (intensity),” “How long I’d feel happy as a student at that college (duration),” “How often I’d feel happy as a student at that college (frequency).” They then rated reliance on forecast unhappiness: “How unhappy I’d feel if I was *not* a student at that college (intensity),” “How long I’d feel unhappy if I was *not* a student at that college (duration),” “How often I’d feel unhappy if I was *not* a student at that college (frequency).”

### Results and discussion

**Fig. 2:**
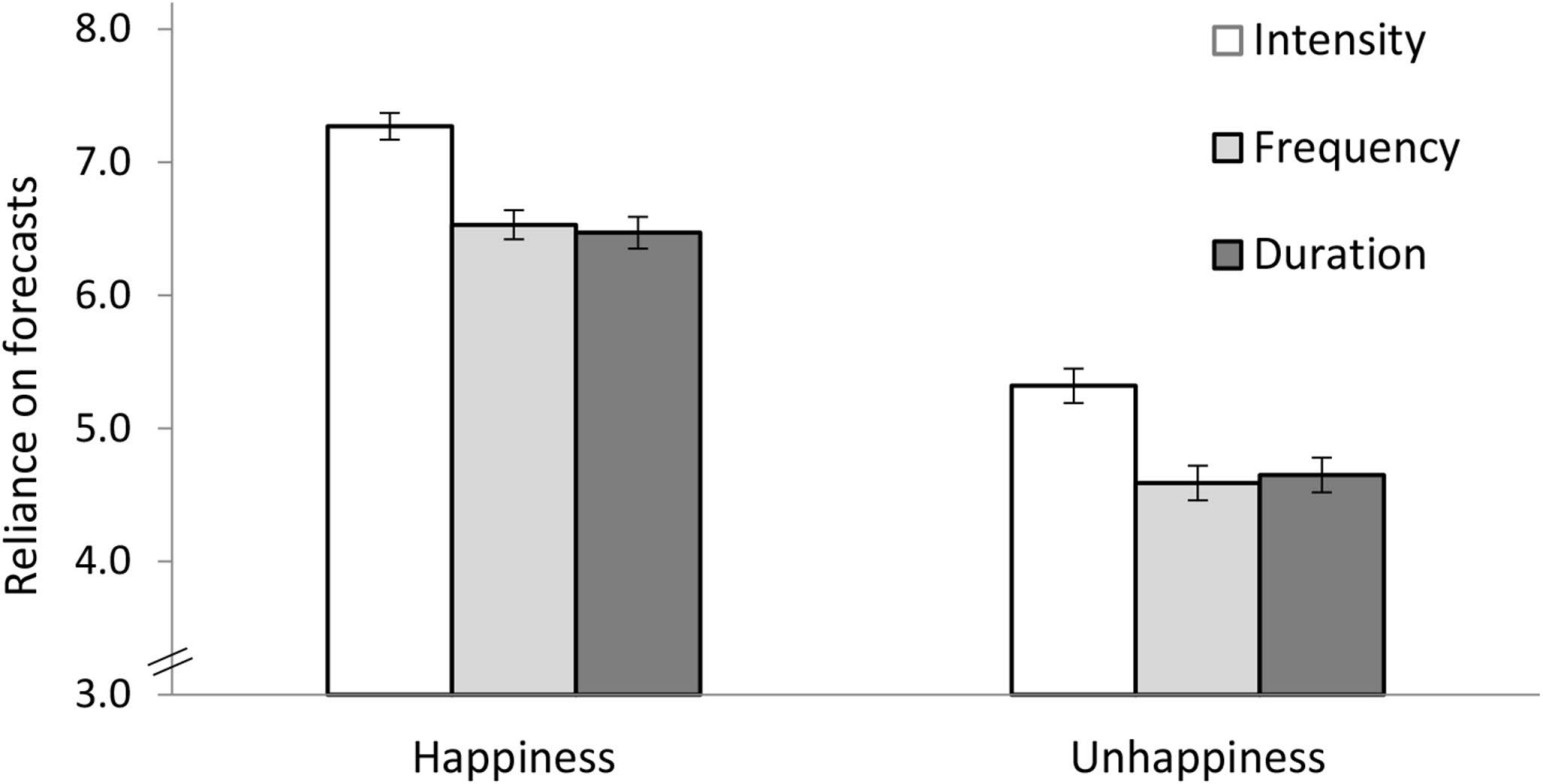
Undergraduates’ Reports of How Much They Relied on Forecasts of Specific Features of Emotion to Decide Which Colleges to Apply to (Study 2) *Note.* Error bars represent ± 1 standard error

To compare how much participants reported having relied on forecasts of specific features of emotion to decide which colleges to apply to, we conducted a repeated measures ANOVA on reliance ratings with emotion feature (intensity, frequency, duration) and valence (happiness, unhappiness) as within-subject variables. The results showed a main effect of feature, *F*(2, 798) = 82.38, *MSE* = 146.58, *p* < .001, η^2^_*p*_ = 0.17. As Fig. 2 shows, participants reported having relied more on forecasts of emotional intensity than frequency, both for happiness, *t*(402) = 8.48, *p* < .001, *d*_*RM, pool*_ = 0.49, 95% CI [0.58, 0.94], and for unhappiness, *t*(401) = 9.16, *p* < .001, *d*_*RM, pool*_ = 0.77, 95% CI [0.57, 0.89]. They also reported having relied more on forecasts of intensity than duration, both for happiness, *t*(402) = 8.06, *p* < .001, *d*_*RM, pool*_ = 0.45, 95% CI [0.60, 0.99], and unhappiness, *t*(401) = 8.35, *p* < .001, *d*_*RM, pool*_ = 0.70, 95% CI [0.51, 0.82]. Reliance did not differ for forecasts of emotion frequency and duration. The results also showed a main effect of valence, *F*(1, 399) = 200.90, *MSE* = 2145.15, *p* < .001, η^2^_*p*_ = 0.33. Participants reported that they relied more on forecasts concerning how happy they would feel as a student in a college than how unhappy they would feel if they were not a student in a college.

Study 2 thus replicated and extended the results of Study 1. To make career and academic decisions with long-term consequences, participants reported having relied more on forecast intensity than frequency or duration, and more on forecast happiness than unhappiness. These findings characterized participants’ forecasts about their emotional experience both when they found out the program they would attend (Study 1) and in the program itself (Study 2).

## Study 3

Studies 1 and 2 assessed the forecasts people reported making to decide among positive outcomes (residency programs and colleges). Study 3 extended the investigation to political decisions which are often motivated by the desire to avoid negative outcomes (Finkel et al., 2020). We assessed how much participants reported having relied on forecasts of the intensity, frequency, and duration of happiness and unhappiness to decide which candidate to vote for in the 2020 U.S. presidential election. We also assessed how accurately participants forecast the intensity, frequency, and duration of their positive and negative feelings about the election outcome.

### Method

**Participants.** Undergraduates (*N* = 309) completed three online questionnaires. As part of a larger project on emotion and decision making, an initial sample of 415 undergraduates completed three online questionnaires: about two weeks before, the week after, and two weeks after, the 2020 U.S. Presidential election. Because Study 3 assessed how participants decided which presidential candidate to vote for, we excluded 105 undergraduates who indicated that they did not vote. We also excluded all participants for whom the sum of three attention check items was more than 4 *SD* below the mean. This resulted in the exclusion of one participant. The sample included undergraduates at large public research universities in California (*n* = 264) and Texas (*n* = 45). Different groups were sampled to capture a range of political preferences. A post hoc power analysis using G*Power targeted a mixed model ANOVA on reliance ratings, testing the interaction of emotion feature and valence as within-subject factors, and vote (Biden vs. Trump or another candidate) as a between-subjects factor. The sample size provided 99% power at 0.05 to detect a small effect (Cohen’s *f*^*2*^ = 0.10). We estimated the effect based on prior findings that ratings of emotional intensity, frequency, and duration tend to be correlated (Sonnemans & Frijda, 1995; Verduyn & Lavrijsen, 2015), likely limiting differences among ratings.

Participants reported their gender as female (78%), male (19%) or other gender identities (3%); the average age was 20.89 years (*SD* = 3.81, range = 18–50). They reported their ethnicity as Hispanic (31%), White (26%), East Asian (15%), Middle Eastern (3%), South Asian (9%), Pacific Islander (4%), Black (2%), or other (10%). Participants received partial course credit for completing the questionnaires. The research was carried out in accordance with the IRBs at the Universities.

**Procedures and materials: Time 1: Two weeks before the election.** Participants completed an online questionnaire about two weeks before the November 3, 2020 presidential election (October 22 - November 2). They forecast the intensity, frequency, and duration of happiness, anger, and fear they would feel days after the election, if Joe Biden won. Participants were instructed to suppose that it is an evening, the week after the outcome of the presidential election is announced, and that Joe Biden won and will be the next president of the United States. They were asked to rate: “How will you be feeling about Joe Biden being elected president? How intensely will you feel [happy / angry / scared],” from 1 *(not at all)* to 9 *(extremely)*. They rated, “How frequently that day will you feel this way about Joe Biden being elected president? [happy / angry / scared]” from 1 *(not at all)* to 9 *(constantly)*. They rated, “Overall, how much of the time that day will you spend in a mood that is [happy / angry / scared]” on a scale from 0% *(not at all)* to 100% *(the entire day)* in 10% increments.

**Time 2: The week of the election**. About one week after the election (November 9–12), the same participants completed a second questionnaire. Using the same prompts and scales as at Time 1, with wording altered to indicate present tense, participants reported the intensity, frequency, and duration of happiness, anger, and fear they were feeling about Biden’s victory. They also indicated whether they had voted and, if so, for whom: Biden, Trump, or another candidate.

**Time 3: Two weeks after the election.** About two weeks after the election (November 16–20), participants completed a third questionnaire. They were instructed: “To help them make decisions, like who to vote for, people sometimes try to imagine or forecast how different outcomes would make them feel. Feelings, like happiness or unhappiness, have several features: Intensity - whether a feeling is gentle or strong. Frequency - whether a feeling occurs rarely or often. Duration - whether a feeling lasts a short or long time.” Participants were then asked how much they relied on forecasts of different features of emotion when they decided which candidate to vote for. Ratings were made on a scale from 1 (*not at all important when I made my decision*) to 9 (*extremely important when I made my decision*).

Specifically, we asked, “When you were deciding who to vote for to be the next President, how important were your forecasts about these features of your future *happiness*? When deciding who to vote for, I thought about:” (1) “How happy I’d feel if the candidate I prefer was elected (intensity)”; (2) “How often I’d feel happy if the candidate I prefer was elected (frequency)”; (3) “How long I’d feel happy if the candidate I prefer was elected (duration)”. Participants then rated the importance of forecasts about features of their future *unhappiness*: (4) “How unhappy I’d feel if the candidate I oppose was elected (intensity)”; (5) “How often I’d feel unhappy if the candidate I oppose was elected (frequency)”; (6) “How long I’d feel unhappy if the candidate I oppose was elected (duration)”. The order in which participants rated forecast intensity, frequency, and duration was randomized for happiness and for unhappiness. The questionnaire used for the overall project was lengthy, so participants also rated three self-report attention check items (e.g., how carefully they read the questions) from 1 *(not at all)* to 9 *(extremely)*.

**Analytic plan.** To analyze the data in Studies 1, 2, and 4, we used repeated measures or mixed model ANOVAs with Type III Sums of Squares, an appropriate approach for moderately unbalanced designs (Smith & Cribbie, 2014). In Study 3, however, the data were sufficiently unbalanced to support the use of linear mixed model analyses (Stroup et al., 2018). The majority of participants voted for Biden (80%), whereas far fewer voted for Trump (17%) or another candidate (3%). Therefore, we used SAS 9.4 PROC MIXED to conduct linear mixed model analyses of: (a) how much participants reported relying on forecasts of different features of emotion, and (b) forecasting accuracy. We used compound symmetry as the covariance structure because all within-subject ratings were made on a uniform scale. (For consistency with the other studies, we also analyzed the Study 3 data using mixed model ANOVAs. The results support the same conclusions reported below and are available online in Supplemental Materials at https://osf.io/3548z/.)

### Results and discussion

In preliminary analyses, we examined the intensity, frequency, and duration of emotion that participants forecast and experienced concerning Biden’s victory. Overall, participants who voted for Biden forecast and experienced strong happiness (means ranged from 5.49 to 7.72) and little anger or fear (range: 1.29 to 2.69). Participants who voted for Trump or another candidate forecast and experienced more moderate feelings of happiness (range: 2.32 to 5.67), anger (range: 3.07 to 4.75), and fear (range: 3.74 to 5.36).

**Fig. 3:**
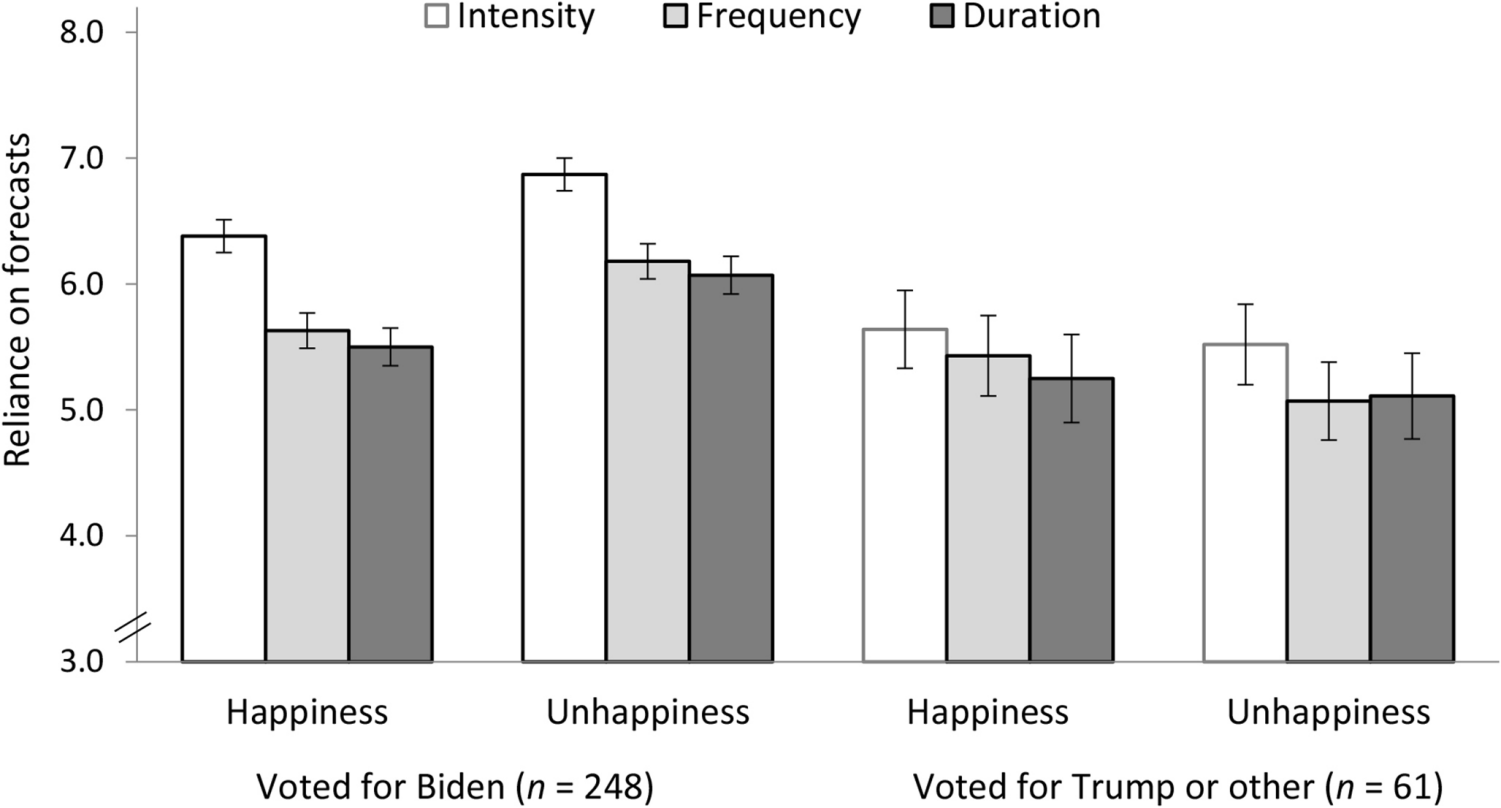
Mean Reported Reliance on Forecasts of Emotional Intensity, Frequency, and Duration to Decide Who to Vote for in the 2020 U.S. Presidential Election (Study 3) *Note.* Error bars represent ± 1 standard error

**Reported reliance on forecasts of specific features of emotion to decide on vote.** We tested the relation of feature (intensity, frequency, duration), valence (positive, negative), and vote (Biden vs. Trump or another candidate) to reliance ratings, using a linear mixed model with emotion feature and valence as repeated factors. A main effect of emotion feature was found, *F*(2, 614) = 17.63, *p* < .001. As Fig. 3 shows, participants reported having relied more on forecasts of intensity than frequency, both for happiness, *t*(308) = 5.94, *p* < .001, *d*_*RM, pool*_ = 0.39, 95% CI [0.43, 0.87], and for unhappiness, *t*(307) = 6.40, *p* < .001, *d*_*RM, pool*_ = 0.47, 95% CI [0.45, 0.84]. They also reported having relied more on forecasts of intensity than duration, both for happiness, *t*(308) = 6.25, *p* < .001, *d*_*RM, pool*_ = 0.37, 95% CI [0.54, 1.04], and unhappiness, *t*(308) = 6.56, *p* < .001, *d*_*RM, pool*_ = 0.46, 95% CI [0.51, 0.94]. Reliance on forecasts of frequency and duration did not differ significantly (*p*s > 0.15).

The results also showed a main effect of vote, *F*(1, 307) = 9.36, *p* = .002, which was qualified by an interaction between vote and valence, *F*(1, 306) = 16.43, *p* < .001. Biden voters reported having relied more on forecasts of how *unhappy* they would feel if the opposing candidate was elected (*M* = 6.38, *SD* = 1.96) than on how *happy* they would feel if their own candidate was elected (*M* = 5.83, *SD* = 1.89), *t*(246) = 4.42, *p* < .001, *d*_*RM, pool*_ = 0.28, 95% CI [0.30, 0.79]. In contrast, those who voted for Trump or another candidate did not differ in the extent to which they reported having relied on forecast unhappiness (*M* = 5.23, *SD* = 2.36) and happiness (*M* = 5.44, *SD* = 2.32), *t*(60) = -0.85, *p* = .40, *d*_*RM, pool*_ = 0.14, 95% CI [-0.68, 0.27].

**Forecasting accuracy.** As in Study 1, we assessed participants’ overall inaccuracy in forecasting different features of emotion by calculating the absolute value of the difference between forecast and experienced emotion. Ratings of anger and fear were moderately to highly correlated when participants reported the intensity, frequency, and duration of emotions forecast (*r*s > 0.65) and experienced (*r*s > 0.58). For parsimony, we used mean ratings of anger and fear in analyses of the accuracy of forecasts of negative emotion. Results of analyses of the direction of forecasting bias for all three emotions are available online in Supplemental Materials, Table S4 (https://osf.io/3548z/).

Using a linear mixed model, we tested the relation of emotion feature (intensity, frequency, duration), valence (positive, negative), and vote (Biden vs. Trump or another candidate) to overall forecasting inaccuracy, with feature and valence as repeated measures. The results, shown in Table 1, revealed a main effect of feature, *F*(2, 611) = 35.27, *p* < .001. Paired *t*-tests, averaging across voter groups and across positive and negative emotion, showed that participants forecast the intensity of their emotional response more accurately (*M* = 0.96, *SD* = 0.83) than its frequency (*M* = 1.43, *SD* = 1.04), *t*(292) = 7.12, *p* < .001, *d*_*RM, pool*_ = 0.34, 95% CI [0.34, 0.60], or duration (*M* = 1.61, *SD* = 1.18), *t*(292) = 9.35, *p* < .001, *d*_*RM, pool*_ = 0.48, 95% CI [0.51, 0.78]. They also forecast the frequency of emotion more accurately than duration, *t*(292) = 2.29, *p* = .02, *d*_*RM, pool*_ = 0.12, 95% CI [0.02, 0.33].

**Table 1:** *Linear Mixed Model Analysis of the Overall Inaccuracy of Forecasts of the Intensity, Frequency, and Duration of Emotion concerning the Outcome of the 2020 U.S. Presidential Election (Study 3)*

Effect	Num DF	Den DF	*F*-Value	Pr > *F*
Between-subjects effects				
Vote (Biden, Trump or other)	1	307	17.55	<.001
Within-subjects effects				
Feature (intensity, frequency, duration)	2	611	35.27	<.001
Emotion (positive, negative)	1	306	28.37	<.001
Feature*Vote	2	611	3.58	0.03
Emotion*Vote	1	306	20.94	<.001
Feature*Emotion	2	591	2.62	0.07
Feature*Emotion*Vote	2	591	4.49	0.01

As Table 1 shows, the linear mixed model results also revealed interactions between emotion feature, valence, and vote. As can be seen in Table 2, participants forecast emotional intensity more accurately than duration regardless of whether they were forecasting positive or negative emotion and regardless of whether or they voted for Biden or another candidate. Participants also forecast emotional intensity more accurately than frequency with two exceptions. For negative emotion, Biden voters directionally forecast intensity more accurately than frequency. For positive emotion, voters for Trump or another candidate directionally forecast intensity more accurately than frequency. However, these differences were not statistically significant.

**Table 2:** *Estimated Marginal Means, and Standard Errors, of the Overall Inaccuracy of Voters’ Forecasts of their Emotional Response to the Outcome of the 2020 U.S. Presidential Election (Study 3)*

		Intensity		Frequency		Duration	
Voter group	Valence	*M*	*(SE)*	*M*	*(SE)*	*M*	*(SE)*
Biden (*n* = 234)	Positive	1.07_a_	(0.09)	1.87_b_	(0.09)	1.88_b_	(0.09)
	Negative	0.69_a_	(0.09)	0.89_a,b_	(0.09)	1.04_b_	(0.09)
Trump/other (*n* = 59)	Positive	1.27_a_	(0.18)	1.47_a_	(0.18)	2.38_b_	(0.18)
	Negative	1.19_a_	(0.18)	1.79_b_	(0.18)	1.96_b_	(0.18)

*Notes.* Overall inaccuracy refers to the absolute value of the difference between forecast and experienced emotion; lower values indicate greater accuracy. Estimated marginal means in a row that lack a common subscript differ significantly at *p* < .05 based on *t*-tests. A few participants did not rate forecast or experienced emotion for every emotion feature, so *n*s varied slightly across means.

In summary, regardless of whether their candidate ended up winning or losing, Study 3 participants reported having relied more on forecasts of emotional intensity than frequency or duration to decide which presidential candidate to vote for. Study 3 also extended Studies 1 and 2 by showing that people do not always report relying more on forecasts of happiness than unhappiness to make decisions. Biden voters reported relying more on forecasts of how *unhappy* they would feel if Trump won the election than on how *happy* they would feel if Biden won. Those who voted for Trump or another candidate did not differ in the extent to which they reported relying on forecast unhappiness versus happiness.

Biden voters may have reported relying primarily on forecast unhappiness to decide their vote because they disapproved of Trump more than they approved of Biden. Indeed, a large-scale poll conducted months before the 2020 election (Knight Foundation, 2020) showed that Biden’s lead among college students was primarily driven by strong aversion to Trump. In this poll, fully 81% percent of students expressed an unfavorable view of Trump, including 68% who reported a very unfavorable view. Students’ support for Biden was more tepid, with only about half of students (49%) expressing a favorable view. Thus, participants reported relying more on forecast unhappiness than happiness when making decisions that were likely motivated by the desire to avoid an aversive outcome.

Finally, whereas Study 1 assessed forecasting accuracy for different features of happiness, Study 3 assessed forecasting accuracy for different features of both negative and positive emotion. The results showed that Biden voters forecast the intensity of their happiness about Biden’s victory more accurately than the frequency or duration of their happiness. Voters for Trump or other candidates forecast the intensity of their negative feelings about Biden’s victory more accurately than the frequency or duration of their negative feelings.

## Study 4

In the first three studies, participants reported how they had made decisions in the past. Because self-reports about past decisions may be subject to retrospective biases (Nisbett & Wilson, 1977), Study 4 assessed how participants would make a future decision with important health implications. We asked participants how they were deciding whether to take a summer trip as Covid-19 rates began to decline. We hypothesized that participants would report relying more on forecast emotional intensity than frequency or duration to decide whether to travel (preregistered at: https://aspredicted.org/CMQ_CMV). Building on Study 3, we also explored whether reported reliance on forecast happiness versus unhappiness would differ for participants who viewed travel during the pandemic as primarily negative versus primarily positive or mixed.

### Method

**Participants.** As part of the same larger project on emotion and decision making as in Study 3, undergraduates at a large public research university in California (*N* = 166) completed an online questionnaire in April of 2021, a time when rates of Covid-19 were declining relative to the peak in January of 2021 (Centers for Disease Control and Prevention, 2021). As in Study 3, we excluded all participants for whom the sum of three attention check items was more than 4 *SD* below the mean. This resulted in the exclusion of one participant. A post hoc power analysis using G*Power showed that for a mixed model ANOVA on reliance ratings, with two within-subject factors (emotion feature, valence) and one between-subjects factor (attitude toward travel), this sample size provided 92% power at 0.05 to detect a small effect (Cohen’s *f*^*2*^ = 0.10). We estimated the effect size based on prior research showing correlations among emotion features (Sonnemans & Frijda, 1995; Verduyn & Lavrijsen, 2015), which would make observing large differences among features unlikely.

Participants reported their gender as female (84%), male (13%) or other gender identities (3%); the average age was 20.88 years (*SD* = 3.58, range = 18–47). They reported their ethnicity as Hispanic (34%), East Asian (20%), White (19%), South Asian (9%), Pacific Islander (4%), Black (1%), or other (13%). Participants received a $5 Amazon gift card for completing the questionnaire. The research was carried out in accordance with the University’s IRB.

**Procedure and materials.** In April of 2021, as rates of Covid-19 were declining, participants completed an online questionnaire which asked them how much they were relying on forecasts of different features of emotion to decide whether to take a summer trip. They were instructed that, “Covid-19 cases are finally declining and more people are vaccinated. As a result, many people are deciding whether to take a trip this summer to spend time with family or friends. To help them make decisions, like whether to take a trip this summer, people sometimes try to imagine or forecast how outcomes will make them feel. Feelings, like happiness or unhappiness, have several features: Intensity - whether a feeling is gentle or strong. Frequency - whether a feeling occurs rarely or often. Duration - whether a feeling lasts a short or long time. The next questions ask how much you will imagine your future feelings in order to decide whether to take a trip this summer to spend time with family or friends.”

Participants then rated how much they would rely on six features of forecast emotion to decide whether to take a summer trip to spend time with family or friends. First, they rated how much they would rely on forecasts about their future *happiness*: “How intensely happy I’ll feel on the trip (intensity)”; “How often I’ll feel happy on the trip (frequency)”; “How long I’ll feel happy on the trip (duration)”. Then they rated how much they would rely on forecasts about their future *unhappiness*: “How intensely unhappy I’ll feel if I don’t go on the trip (intensity)”; “How often I’ll feel unhappy if I don’t go on the trip (frequency)”; “How long I’ll feel unhappy if I don’t go on the trip (duration)”. The order in which participants rated reliance on forecast intensity, frequency, and duration was randomized for happiness and for unhappiness.

To assess whether participants viewed travel during the pandemic as positive or negative, they rated their agreement with two statements from 1 *(strongly disagree)* to 9 *(strongly agree)*: “Due to the global pandemic, I’ll be especially happy to have a chance to take a trip this summer to spend time with family or friends” (positive), and “My ongoing worries about Covid-19 will make it difficult to feel happy on a trip this summer to spend time with family or friends” (negative). Finally, they rated three self-report attention check items from 1 *(not at all)* to 9 *(extremely)*.

### Results and discussion

Participants expressed moderately strong agreement that, due to the pandemic, summer travel was both a source of happiness (positive: *M* = 5.55, *SD* = 2.59) and a source of worry (negative: *M* = 5.34, *SD* = 2.70). Using these ratings, we grouped participants based on whether they viewed travel as: (a) primarily positive, or equally positive and negative (64%), or (b) primarily negative (36%). Results did not differ from those reported when attitudes toward travel were coded using three categories: primarily positive, equally positive and negative, and primarily negative.

**Fig. 4:**
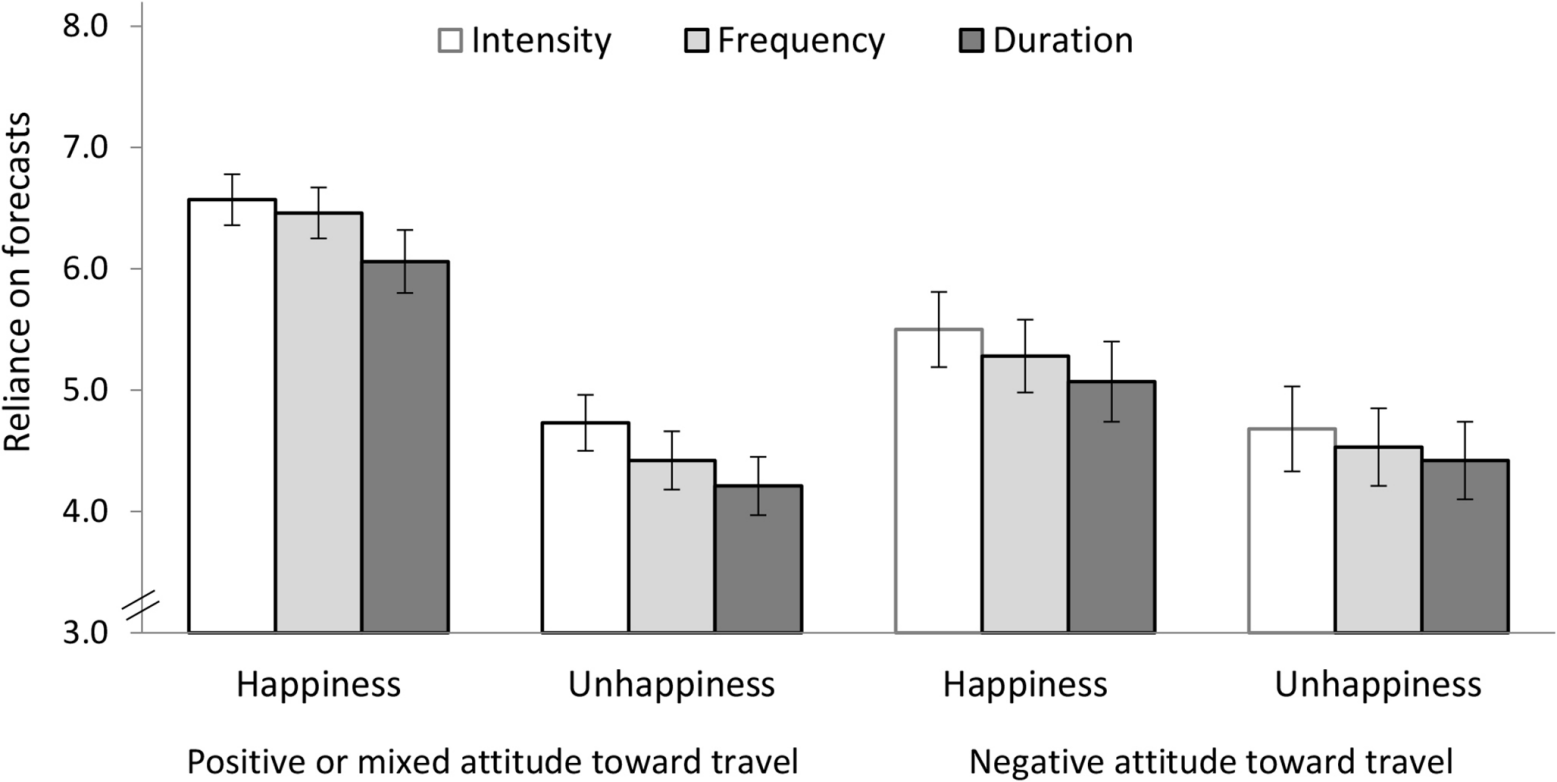
Participants’ Reports of How Much They Would Rely on Forecasts of Emotional Intensity, Frequency, and Duration to Decide Whether to Travel as Covid-19 Rates Declined (Study 4) *Note.* Error bars represent ± 1 standard error

Figure 4 shows how much participants reported relying on different features of emotion to decide whether to take a summer trip during the Covid-19 pandemic. We conducted a mixed model ANOVA on reliance ratings with emotion feature (intensity, frequency, duration) and valence (happiness, unhappiness) and as within-subject variables, and with attitude toward travel (positive or mixed vs. negative) as the between-subjects variable. A main effect of feature was found, *F*(2, 326) = 7.08, *p* = .001, η^2^_*p*_ = 0.04. Overall, to decide whether to take a summer trip, participants reported relying more on forecast emotional intensity (*M* = 5.45, *SD* = 1.94) than frequency (*M* = 5.25, *SD* = 1.91), *t*(162) = 1.98, *p* = .049, *d*_*RM, pool*_ = 0.20, 95% CI [0.0002, 0.42], or duration (*M* = 4.99, *SD* = 2.06), *t*(162) = 3.85, *p* < .001, *d*_*RM, pool*_ = 0.38, 95% CI [0.23, 0.71]. Participants also reported relying more on forecast frequency than duration, *t*(162) = 2.38, *p* = .02, *d*_*RM, pool*_ = 0.26, 95% CI [0.04, 0.47].

A main effect of valence was also found, *F*(1, 163) = 40.86, *p* < .001, η^2^_*p*_ = 0.20. Across features, participants reported relying more on forecasts of how happy they would feel if they traveled (*M* = 5.96, *SD* = 2.16) than how unhappy they would feel if they did not travel (*M* = 4.49, *SD* = 2.26). However, this effect was qualified by an interaction between the valence of forecasts and attitude toward travel, *F*(1, 163) = 7.95, *p* = .005, η^2^_*p*_ = 0.05. As Fig. 4 shows, participants reported relying more on forecast happiness if their attitude toward traveling during the pandemic was positive or mixed than if their attitude was negative, *t*(163) = 3.12, *p* = .002, *d* = 0.51, 95% CI [0.39, 1.73]. Participants’ reported reliance on forecast unhappiness did not differ significantly as a function of their attitude toward travel, *t*(163) = 0.28, *p* = .78, *d* = 0.04, 95% CI [-0.62, 0.83].

Thus, extending the first three studies, in which participants reported how they had made decisions in the past, Study 4 assessed how participants would make a future decision with important health implications. To decide whether to take a summer trip in the future as rates of Covid-19 were declining, participants reported that they would rely more on forecasts concerning the intensity of emotion than the frequency or duration of emotion. Participants also reported that they would rely more on forecasts of how happy they would feel if they traveled than how unhappy they would feel if they did not travel. However, reported reliance on forecast happiness was greatest for participants who viewed traveling during the pandemic as positive or mixed rather than negative.

### General discussion

To make decisions, people forecast how the outcomes of their choices will make them feel. This investigation was the first to examine the specific features of emotion people report forecasting to make decisions. Across four studies, participants reported relying more on forecast emotional intensity than frequency or duration to guide decisions about their careers, education, politics, and health. In Studies 1 and 3, we also assessed the accuracy of participants’ forecasts. Participants in both studies forecast the intensity of emotion more accurately than frequency or duration. Moreover, in Study 1, graduating medical students’ accuracy in forecasting the intensity of their feelings about being matched with a particular residency program uniquely predicted greater satisfaction with their program months later. The focus of research on affective forecasting has been on understanding causes and consequences of bias. We found, however, that the feature of emotion which people perceive to be driving their decisions has the added value of being the feature they forecast most accurately. These findings contribute to affective forecasting theory by showing that emotional intensity forecasts are both valued and valuable when people are making decisions of lasting importance for their lives.

### Medical students report relying on forecast emotional intensity to make decisions

Match Day is the culmination of students’ years in medical school. The residency programs with which graduating students are matched have implications that range from whether they will receive three to seven years of training in their preferred specialty to whether they will live close to or distant from family and friends. Students’ decisions about how to rank residency programs in preparation for the match play an important role in determining the outcome. In Study 1, graduating medical students reported having relied more on forecasts of emotional intensity than frequency or duration to decide how to rank residency programs. Medical students’ reported reliance on forecast intensity appears to have been warranted. They forecast how intensely happy they would feel after matching with a specific residency program more accurately than the frequency or duration of their happiness. As a group, they showed no significant over- or underestimation in forecasting the intensity of their happiness but overestimated its frequency and duration. This investigation thus joins a growing body of research showing that, although people can over- or underestimate the intensity of future feelings, they forecast emotional intensity more accurately than frequency, mood, or feelings in general (Doré et al., 2016; Lench et al., 2019; Levine et al., 2012; for a review, see Levine et al., 2018). Moreover, medical students’ accuracy in forecasting emotional intensity uniquely predicted greater satisfaction with their programs during residency. These findings suggest that forecast emotional intensity is a salient and relatively accurate index of how closely decision outcomes align with people’s goals.

### Follow-up studies

Building on Study 1, three follow-up studies assessed the features of emotion people reported forecasting to make other important decisions. In Study 2, to decide which colleges to apply to, undergraduates reported having relied more on forecasts of the intensity, than the frequency or duration, of their emotional experience as a student. In Study 3, to decide which presidential candidate to vote for, participants reported having relied more on forecasts of emotional intensity than frequency or duration. Participants also forecast the intensity of their feelings about Biden’s victory more accurately than their frequency or duration. Whereas the first three studies focused on decisions in the recent past, Study 4 assessed how participants were currently deciding on future plans that could impact their own health or that of family or friends. To decide whether to travel to spend time with family or friends as rates of Covid-19 were declining, participants reported that they would rely more on forecasts of emotional intensity than frequency or duration.

People have limited ability to introspect about their thoughts and feelings (Nisbett & Wilson, 1977). Can they really distinguish among features of their emotional experience? In this investigation, we carefully defined each feature of emotion for participants. We explained, for instance, that just as a musical note in a song can be loud or soft, occur more or less frequently, and last for a longer or shorter period of time, emotions vary in their intensity, frequency, and duration. Each questionnaire item explicitly noted the specific feature of emotion that participants were asked to rate. Importantly, in Study 1, analyses of correlations among medical students’ judgments provided evidence that they did make distinctions among emotion features. Across students’ reliance judgments, affective forecasts, and reports of their emotional experience, only about half of the variance in participants’ reports of one emotion feature could be explained by other emotion features (see Lench et al., 2019 for similar results). Affective forecasting studies rarely compare judgments about the intensity, frequency, and duration of emotion (Levine et al., 2012). Yet our findings show that people distinguish among these emotion features, they forecast intensity more accurately than frequency or duration, and they report relying more on the forecasts that are relatively accurate to make decisions.

### Reported reliance on forecast happiness versus unhappiness to make decisions

We also compared how much participants reported relying on forecasts about how happy they would feel if they attained a desired outcome versus how unhappy they would feel if they did not attain a desired outcome. People tend to be optimistic when imagining their futures (Barsics et al., 2016; Lench et al., 2021; Sharot, 2011). Likewise, participants reported relying more on forecast happiness than unhappiness to rank residency programs (Study 1), to decide which colleges to apply to (Study 2), and to decide whether to travel as Covid-19 infections declined (Study 4). But exceptions to this optimistic tendency were found. In Study 3, to decide which presidential candidate to vote for, Biden voters reported having relied more on forecasts of how *unhappy* they would feel if Trump was elected than how *happy* they would feel if Biden was elected. Voters for Trump or another candidate reported having relied as much on forecast unhappiness as happiness. Consistent with large scale polls of the political views of college students (e.g., Knight Foundation, 2020), Biden voters may have reported relying more on forecast unhappiness because they disapproved of Trump more than they approved of Biden. In Study 4, participants who viewed travelling during the pandemic as primarily negative reported relying less on forecast happiness than those whose attitude toward travel was positive or mixed.

Taken together, these findings suggest that people look to relevant emotions to guide decisions about outcomes they consider to be good or bad. They focus on how happy outcomes will make them if they anticipate pursuing opportunities (e.g., career and educational training), but focus on how unhappy outcomes will make them if they anticipate forgoing opportunities or avoiding threats (e.g., voting against a greatly disfavored political candidate). Strikingly, regardless of whether they were anticipating future happiness or unhappiness, people reported relying more on forecast emotional intensity than frequency or duration to make decisions.

### The perceived value and the accuracy of emotional intensity forecasts

Why do people report relying on forecasts of the intensity of emotion to make decisions? Why are intensity forecasts more accurate than other forecasts? When forecasting how future events will make them feel, people draw on their evaluations of similar past events (Morewedge et al., 2005). These evaluations are heavily informed by peak intensity which reflects the importance of events (Ariely & Loewenstein, 2000; Fredrickson, 2000; Frijda et al., 1992; Levine et al., 2020). Failure to encode how long past emotional experiences lasted may make people less likely to forecast the frequency and duration of future emotion. Thus, people may rely on forecast intensity to make decisions because this feature of emotion is highly salient and indicates the importance of decision outcomes for their goals.

With respect to accuracy, people focus on central, goal-relevant characteristics of events both when forecasting how they will feel (Wilson et al., 2000) and when experiencing intense emotion (Levine & Edelstein, 2009). This common focus of attention on the core aspects of events may promote more accurate forecasts (Levine et al., 2012, 2018). Moreover, the relevance of events for people’s goals remains comparatively stable over time (McAdams & Olson, 2010). In contrast, people’s thoughts and regulatory processes shift rapidly over time, are harder to anticipate, and often diminish the frequency and duration of emotion. As a result, people often overestimate these features of future emotion (Gilbert et al., 1998; Lench et al., 2019; Wilson et al., 2000).

### Limitations and future research directions

Although our findings provide new insights on the forecasts people perceive to be valuable for guiding their decisions, limitations of the studies should be noted. First, our ability to draw causal inferences (such as the relation between forecasting accuracy and satisfaction with residency programs in Study 1) is limited by the correlational nature of the data. Second, people may be inaccurate in their reports concerning the features of emotion that guide their decisions. In future research, investigators could assess how priming participants to forecast different features of emotion influences the quality of decisions and satisfaction with decision outcomes.

A third limitation is the use of retrospective reports in some studies. Participants reported the features of emotion they had previously forecast to rank residency programs, select universities to apply to, and decide who to vote for. This timing was deliberate. It allowed participants to reflect on how they made their choices and, importantly, avoided ethical concerns that study questions might influence life-altering choices. However, self-reports may be subject to retrospective biases (Nisbett & Wilson, 1977). For example, people’s reports about how they made their choice could vary depending on whether they had experienced a positive or negative outcome.

We took several steps to address this: In Study 1, medical students reported how much they relied on forecasts of different features of emotion to rank residency programs before they learned which program they were matched with. This removed the possibility that their reports could be influenced by the outcome of the match. In Study 3, we compared the reports of participants for whom the election outcome was positive versus negative. In Study 4, we asked participants how they would make a *future* decision about whether to travel during the pandemic. Across studies, participants reported relying more on forecast emotional intensity than frequency or duration to make decisions regardless of whether the outcome was unknown or known, positive or negative, and regardless of whether the decision was made in the past or would be made in the future. Thus, retrospective bias does not appear to account for participants’ reports that they relied most on forecast emotional intensity.

Emotional intensity, frequency, and duration are typically moderately correlated. For instance, people continue to think about and elaborate on the self-relevance of events that evoke intense emotion, causing those emotions to endure (Frijda et al., 1992; Verduyn et al., 2015). Thus, accurately forecasting the intensity of emotion often provides valuable insight into how frequent and lasting the emotion will be. But not always. Wisdom may entail discriminating outcomes that will elicit intense but short-lived happiness (e.g., addictive drug use, big ticket purchases) from those that will bring about frequent and lasting happiness (Bornstein & Pickard, 2020; Diener et al., 2009; Jachimowicz et al., 2021). Thus, future research should also explore conditions under which forecasting the frequency and duration of emotion leads to better decisions than forecasting the intensity of emotion.

Finally, future research should examine whether the forecasts people report relying on to make decisions vary across individuals, age groups, and cultures. For instance, people who regulate anxiety using defensive pessimism may rely more on forecast negative than positive emotion in order to avoid future threats (Hazlett et al., 2011).

## Conclusion

Research on affective forecasting has focused primarily on when and why forecasts are biased, and on the costs and benefits of bias for decision making. Overestimating the emotional impact of decisions can motivate goal pursuit (Morewedge & Buechel, 2013), but it can also lead people to squander their efforts, pursuing outcomes that do not make them as happy as anticipated, and avoiding ones that do not make them as unhappy as anticipated (Gilbert et al., 2002). In this investigation, we took a new approach by examining the features of emotion people report forecasting to guide their decisions. Whether deciding how to rank medical residency programs in preparation for being matched with a program, which colleges to apply to, which presidential candidate to vote for, or whether to travel as rates of Covid-19 declined, people reported that they had relied, or would rely, more on forecasts of emotional intensity than frequency or duration. This perceived reliance on forecast intensity appears to have been warranted. Participants forecast emotional intensity more accurately than frequency or duration. Moreover, in Study 1, medical students’ accuracy in forecasting emotional intensity was uniquely associated with greater satisfaction with their residency programs several months later. People’s reported reliance on, and relatively accurate prediction of, emotional intensity when making life-changing decisions provides important new evidence of the adaptive value of affective forecasts.

## Data Availability

National Science Foundation Award #1,451,214 to Linda J. Levine and Award #1,451,297 to Heather C. Lench supported data collection for Study 1 as part of a larger project on emotion and decision-making. Data and software code for all studies in this paper have been made publicly available at the Open Science Framework (OSF) and can be accessed at https://osf.io/3548z/. Study 4 hypotheses were preregistered at: https://aspredicted.org/CMQ_CMV.
